# Trajectories of depression and anxiety in adults with rare disorders across 13 months during the COVID-19 pandemic

**DOI:** 10.1186/s13023-025-03633-3

**Published:** 2025-03-20

**Authors:** Øyvind Halsøy, Stian Orm, Hugo Cogo-Moreira, Wendy K. Silverman, Krister Fjermestad

**Affiliations:** 1 Frambu Resource Center for Rare Disorders, Siggerud, Norway; 2 Division of Mental Health Care, Innlandet Hospital Trust, Brumunddal, Norway; 3 Department of Education, ICT and Learning, Østfold University College, Fredrikstad, Norway; 4 School of Medicine, Yale University, New Haven, USA; 5 Department of Psychology, University of Oslo, Oslo, Norway

**Keywords:** Rare disorders, Adults, Mental health, Covid fear, Longitudinal studies

## Abstract

**Background:**

Adults with rare disorders experience multiple psychosocial risk factors beyond their medical symptoms, including impaired quality of life, social isolation, loneliness, and mental health problems. These risk factors were amplified during the COVID-19 pandemic, when health care appointments and social/vocational activities were reduced or cancelled. There is a lack of longitudinal data tracking this population over time, making the long term consequences uncertain.

**Methods:**

We conducted a monthly survey of 58 adults aged between 19 and 71 years (*M* = 45.1 years, *SD* = 12.6) with rare disorders across 13 months during the COVID-19 pandemic in Norway. We measured symptoms of anxiety and depression with the Hopkins Symptom Checklist-5. Covid fear was measured with the Coronavirus Anxiety Scale. We examined the mental health and covid fear trajectories across the 13 months with multi-level growth curve models with repeated measures at Level 1 and individuals at Level 2. To account for differences in governmental restrictions throughout the 13 months, we used the *stringency index* from The Oxford Covid-19 Government Response Tracker.

**Results:**

The growth models indicated stable levels of anxiety and depression over 13 months that were elevated compared to existing population data and were unpredicted by pandemic restrictions. The level of covid fear was significantly associated with the levels of anxious and depressive symptoms.

**Conclusions:**

The current study found elevated and stable trajectories of mental health symptoms throughout the pandemic for persons with rare disorders. This highlights the necessity of investigating the long-lasting influence of the pandemic on mental health among individuals with rare disorders.

## Introduction

Most countries implemented interventions to eliminate the spread of the COVID-19 pandemic. These measures often included physical distancing policies, lockdowns, and prompting a shift to virtual life [1]. These restrictions changed the structure of everyday life for all individuals, and created a set of unique challenges for people with rare disorders [2]. Health care appointments were cancelled, and vocational activities were reduced [3, 4] Rare disorders are disorders that affect less than 1:2000 individuals [5]. More than 7000 rare disorders have been identified globally, and even though each separate disorder is rare, up to about 6–8% of the population suffer from at least one rare disorder [6]. Rare disorders are often chronic and require sustained contact with health care providers throughout the entire lifespan [6]. This results in a high burden on subjective wellbeing [7] as well as a strain on healthcare services.

Rare disorders show high symptom heterogeneity, however people with rare disorders often share common factors which have been tied to increased risks for anxiety and depression [8, 9]. These include risk factors related to the somatic aspects of the diagnosis, but also challenges related to structural barriers in society [10, 11]. Somatic risk factors include multi-morbidity, and frequent and complex somatic issues [9, 10], while structural risk factors include difficulties in navigating health care services, lack of information about the diagnosis, experiences of prejudice, and economic challenges [8]. Both somatic and structural risk factors have been found to increase the risk of symptoms of anxiety and depression [9].

Previous research has indicated that individuals with rare disorders often have worries related to their own physical health, which could lead to more anxiety tied to an infection of COVID-19. They have lower levels of trust in the healthcare system, as their primary care providers may have limited knowledge of their condition, and they may feel unsupported in navigating their diagnosis [7].

Additionally, rare disorders are frequently diagnosed years after the onset of symptoms, which may make persons with rare disorders feel insecure about the health system [7]. In sum, the challenges in trusting healthcare professionals and the periods of diagnostic uncertainty can lead to more fear and worry about health in persons with rare disorders.

### Psychosocial risk factors and the pandemic

Several risk factors were intensified for people with rare disorders during the pandemic. Several studies have found correlational and prospective evidence for the number of stressful life events and heightened levels of stress to increase risk of developing mental health problems in people with rare disorders [12]. During the pandemic, health care appointments were cancelled, vocational activities were reduced, many lost their jobs, many received conflicting advice about the severity of an infection, and social interaction were limited [4, 5, 13]. Although not specifically related to rare disorders, a study from the British Birth cohorts [14] found long standing illness to be a significant predictor of increased depressive symptoms. In addition, generational differences in mental health symptoms were observed, indicating age to be an important factor for mental health problems during the pandemic [14].

In a cross-sectional study we found evidence of a heightened level of mental distress in individuals with rare disorders compared to norms [15]. The initial study found that fear of being infected by COVID-19 was a significant risk factor for symptoms of anxiety and depression. The cross-sectional nature of our initial study made it difficult to determine whether increased levels of anxiety and depression were a temporary reaction to the public health crisis, and if their mental health would improve or deteriorate over time.

In the general population, [16, 17] found fluctuations in symptoms of anxiety and depression throughout the stages of the pandemic, with more severe symptoms during periods of more restrictions and higher spread of the virus. Rosa et al., found levels of depression to decrease from the first lockdown in May 2020, to September 2020, when the restrictions were eased, and increasing again in the second lockdown in February/March 2021. These finding suggest that the strictness of the restrictions covary with mental health problems. Shevlin et al. [18] found significant heterogeneity in trajectories throughout the pandemic in the general population. Some individuals exhibited stable low or stable high levels of anxiety and depressive symptoms, whereas subgroups were identified that displayed either initially high and thereafter declining symptoms or initially low and thereafter increasing symptoms. Findings based on general population samples may not be generalizable to individuals with rare disorders, therefore it is important to understand how people with rare disorders fared throughout the pandemic to inform post-pandemic services and prepare services for returning pandemics.

### Research questions and hypotheses

We address two research questions in the current study. (1) How did the trajectories of mental health symptoms in people with rare disorders evolve throughout 13 months of the pandemic?; and (2) How did covid fear influence mental health problems in people with rare disorders over this 13-month interval of the pandemic? Based on our earlier findings [15], we expected levels of anxiety and depressive symptoms to be higher in stages of the pandemic with more restrictions, we expect higher fear of covid to be associated with more mental health symptoms over time, and we expect older individuals to have less symptoms of mental health problems due to a more stable life situation.

## Methods

### Participants and procedures

The participants were recruited through the social media channels of Frambu Resource Center for Rare Disorders, a national resource center for rare disorders in Norway. To be eligible for inclusion the participants needed to be diagnosed with a rare disorder. No financial compensation was offered for study participation. The study was approved by the local review board for research ethics.

We followed participants monthly for 13 months. The data were collected from 27.07.2020 to 09.11.2021 and comprised one standardized questionnaire delivered monthly. The questionnaires were completed online, in their own residence. Participants were recruited continuously, meaning that the participants answered the questionnaires at different dates, but with the same interval of one month between each measurement. The participants (N = 58) were aged between 19 and 71 years (*M* = 45.1 years, *SD* = 12.6) and had Norwegian as their first language. Participants had a wide range of diagnoses. More than 20 rare neurodevelopmental disorders were represented in the sample. The disorders with more than one participant included Charcot-Marie-Tooth disease, Morbus Osler disease, Hypogammaglobulinemia, Henoch-Schönlein purpura, Neurofibromatosis type 1, Ehlers-Danlos syndrome, Limb-Girdlemuscular dystrophies, and common variable immunodeficiency.

### Measures

#### Demographic information

The participants were asked about their main source of income, their age, their diagnosis, whether that had been diagnosed with the COVID-19 in the last 30 days, about their social interactions, and about which services they were receiving from the public health care system. The study was conducted in Norway.

#### Anxiety and depression

The Hopkins Symptom Checklist (SCL-5; 19)was used to measure depressive and anxiety symptoms. This self-report measure includes five questions to assess psychological distress, mainly symptoms of anxiety and depression. Participants were asked how affected they have been from the following symptoms during the last week: “Nervousness and agitation, feeling scared or anxious, feel hopeless towards the future, Feeling Down, and Worrying” using a scale from 0 to 4, (0: Not at all, 4: Extremely). Scores are calculated by taking the mean value of responses, and scores are interpreted as a global severity index ranging from 1 to 4. A cutoff of 1.85–2.0 on the global severity index has been suggested, 50–60% of the cases identified using this cutoff, is expected to fulfill the criteria for one or more mental disorders in a clinical interview [17]. The SCL-5 has demonstrated convergent validity with other measures of mental health, including longer versions of the SCL (r’s 0.76–0.97; Strand et al. 2003). This validation however has only been completed in the general population, and there are currently no examinations of validity in people with rare disorders. In the current study, the SCL-5 had a Cronbach’s α > 0.90 for all time points. The official Norwegian version of the SCL-5 was used [19].

#### COVID-19-related anxiety

The Coronavirus Anxiety Scale (CAS;20) was used to measure *COVID-19 related anxiety*. This self-report measure includes four items to assess anxiety symptoms related to COVID-19. Participants were asked how affected they have been from the following fears related to the corona virus during the 14 days: Feeling dizzy or lightheaded, lost interest in eating, frozen or paralyzed, or nausea and stomach pains. These questions are rated on a scale from 0 to 5 (0: Not at all, 5: Almost every day). The CAS have shown to have excellent reliability in the original study by [20]. Furthermore replications by [21, 22] have also demonstrated excellent reliability. The CAS was translated to Norwegian by one of the authors of this paper and a backtranslation was approved by the scale’s developer. The CAS had an acceptable reliability in our study, Cronbach’s α = 0.74.

#### Pandemic restrictions

The intrusiveness of the covid-related restrictions changed throughout the pandemic. To account for this, we used the *stringency index average* from The Oxford Covid-19 Government Response Tracker. The stringency index is a measure based on nine response indicators including school closures, workplace closures, and travel bans, ranging from 0 to 100 (100 = strictest). Our examination specifically considered the data for Norway among the 180 countries covered. [1].

#### Data analytic plan

We used Bayesian Multilevel Mixed effects models to analyze the growth curves of the repeated measures. Individual participants were chosen as the level 2, and each repeated measure was treated as level 1. We built models in a sequential order, starting with the simplest models before adding more complexity. We first fit an unconditional model with random intercepts only, with no predictors. Secondly, we compared this model to a model with random slopes for each participant, meaning that random slopes were added as a random effects and time as a fixed effect. We then added our theoretically derived predictors: Age & COVID-19 related anxiety. Lastly, we added an interaction between time and Pandemic Restrictions. Interaction between time and Stringency Index was explored because not all respondents answered the questionnaire at the same dates. Since the restrictions were subject to change, we wanted to control for varying degree of strictness of restrictions at every time point. Age and COVID-19 Anxiety were mean centered, meaning that the intercept represents the expected value when Age, COVID-19 anxiety are at their mean, and time is 0 (the start of the pandemic). Priors were weakly informative based on population estimates by [19]. The prior for the intercept was a normal distribution with a mean = 1,3 and SD = 1, the prior for time was a normal distribution with mean = 0, and SD = 1, the prior for sigma, (the error-variance) was exponential, 0.2.

We selected the model that best represented our data based on theory and information criteria. The criteria for model comparisons were leave-one-out information criteria (LOO-IC) and Watanabe–Akaike information criteria (WAIC). For Bayesian models, a general model selection strategy is comparing the difference between the expected logpredictive density (ELPD) estimates. Models were concluded to be better fitting if the ELPD is larger than four and more than double the magnitude of its standard error [23]. Convergence were determined by examining trace-plots and all r-hat values were < 1.05.

Longitudinal surveys containing repeated measures often have missing data, and these were assumed to be missing at random. To account for missing data Model Based imputation was used, making it possible to analyze data from participants who had not responded at every wave [24]. Model based imputation is a more recent development in handling missing data and has shown to be an improvement in handling complex missing data such as multilevel models with random effects [25]. Ten imputed datasets were created in the BLIMP software, and models were fit in R, using the Brms package [26].

## Results

At baseline the participants had a mean global severity index of *M* = 2.24 (*SD* = 0.92) on the SCL-5. A cutoff of 1.85–2.0 on the global severity index has been suggested, 50–60% of the cases identified using this cutoff, is expected to fulfill the criteria for one or more mental disorders in a clinical interview [17]. Using a cutoff of 2.0, 33 out of 58 participants score above the cutoff at baseline.

### Results from the mixed model

Our best fitting model included a random intercept and a random slope. This indicates a significant variation between individuals in the levels of mental health problems at baseline and that there was significant heterogeneity in individual trajectories throughout the pandemic. Age, time, and *COVID-19 related anxiety* were included as covariates. A model with an interaction effect between time and pandemic restrictions were tested but rejected based on information criteria.

### Results for research question 1

**Fig. 1:**
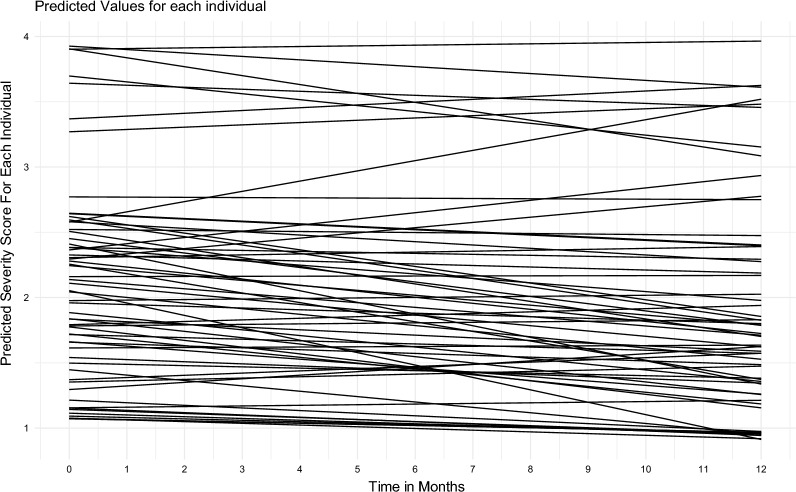
A plot with predicted growth curves for every participant in the study

**Fig. 2:**
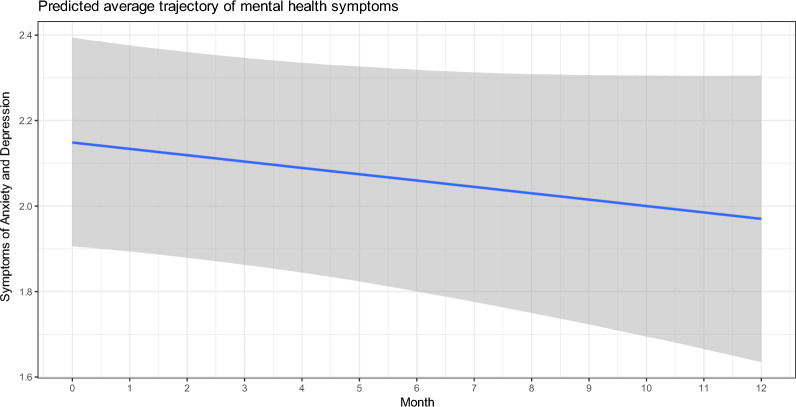
The average trajectory of mental health symptoms across 13 months with 95% CI around the predicted mean trajectory:

The effect of time was estimated to be β = − 0.02, (s.e. = 0.01, 95% C.I [− 0.07, 0.02]), indicating that the average trajectory throughout the pandemic is around 0, suggesting a stable and flat trend throughout the pandemic. Furthermore, we examined an interaction effect between time and pandemic restrictions, β = 0.01, (s.e. = 0.02, 95% C.I [− 0.03, 0.04]), indicating that periods with stricter restrictions were not different from periods without strong restrictions (Figs. 1, 2, Table 1).

**Table 1:** Sociodemographic characteristics of the 58 participants at baseline

Baseline characteristic	*n*	%	Mean	SD
Age (years)			45.1	12.6
Income				
Employed	28	48.3		
Sick Leave	2	3.4		
Disability Benefits	24	41.3		
Retired	3	5.2		
No income	1	1.7		
Contact last 30 days with				
Family	55	94.8		
Friends	51	87.9		
General Practitioner	32	55.1		
Specialist health service	24	41.3		

### Results for research question 2

*COVID-19 related anxiety* was related to increased mental health problems β = 0.10, (s.e. = 0.02, 95% C.I [0.05, 0.15]), indicating that individuals who experience more fear related to the virus had more symptoms of anxiety and depression at baseline. The effect of age on mental health symptoms was 0, β = − 0.02, (s.e. = 0.02, 95% C.I [− 0.04, 0.0]), indicating age did not predict differences in symptoms of anxiety and depression at baseline. The regression coefficients for all models, including is available in Table 2.

**Table 2:** Results from the multi level mixed effects model

Term	*β*	Std.Error	95% CI
Intercept	2.23	0.16	[1.92, 2.54]
Time	− 0.02	0.02	[− 0.07, 0.02]
Restrictions	− 0.02	0.09	[− 0.20, 0.15]
Age (mean centered)	− 0.02	0.01	[− 0.04, 0.00]
COVID Anxiety (mean centered)	0.10	0.02	[0.05, 0.15]
Time:Restrictions	0.01	0.02	[− 0.03, 0.04]
sd_id__Intercept	0.86	0.09	[0.70, 1.07]
sd_id__time	0.06	0.01	[0.04, 0.08]
cor_id__Intercept__time	− 0.09	0.20	[− 0.47, 0.32]
sigma	0.22	0.02	[0.18, 0.27]
nu	2.4	0.50	[1.69, 3.67]

## Discussion

The aim of the study was to examine trajectories of anxiety and depression throughout 13 months of the COVID-19 pandemic in individuals with rare disorders. We hypothesized that different periods of the pandemic would be associated with fluctuations in symptoms of anxiety and depression. Based on previous studies finding increased symptoms of anxiety and depression during lockdowns, we hypothesized that periods containing more intrusive restrictions would lead to increased symptoms. These periods lead to increased isolation from family and friends and meant that healthcare was more difficult to access. Furthermore, the strictest lockdowns were typically found during the early part of the pandemic, thus we expected the gradual re-opening of society to be associated with an improvement of symptoms of anxiety and depression. The increased use of telehealth, and other digital solution were hypothesized to ease the isolation and improve mental health symptoms.

In our findings individuals with rare disorders followed a longitudinal trajectory with stable high levels of mental health symptoms. While not relating to rare disorders specifically a study by Shevlin et al. [18], which used chronic disease as a predictor in a latent growth class membership found people with chronic diseases had an odds ratio of 2.87 for a trajectory charachterized by stable and high levels of mental health symptoms. One way to interpret these findings might be that people with somatic issues might still stay at home even after the restrictions are lifted in fear of getting infected. A study by Kaya et al., 2021 found that people with chronic illness were less likely to go to the hospital even in an emergency than controls [27].

Stable high levels of mental distress throughout the 13 months might be explained by the increased risk factors that many people with rare disorders experienced during the pandemic. In a survey of people with rare disorders, 83% of respondents reported that the covid pandemic had disrputed their contact with healthcare, 60% had their psychiatric follow up interupted, 70% had medical appointments cancelled or postponed, and 9% of participants reported this disruption of medical care to probably be life threatening [3]. People with rare disorders also report lower levels of trust in health care providers, which could lead to more fear and worry regarding their own health, and a stronger use of unreliable sources of information, increasing their levels of anxiety [7, 28].

A plausible interpretation for COVID-19 anxiety being related to higher levels of mental health symptoms could be that people who have more *COVID-19 related anxiety* are related to dispositional anxiety which assumes that some individuals are more prone to experiencing negative emotions. Especially since we did not find an interaction between time and restrictions, this might indicate that some individuals are more vulnerable to experiencing distress. Another interpretation is that the relationship between COVID-19 related anxiety and mental health symptoms reflects a more serious underlying medical conditions. Where people who have a more serious underlying condition have more fear of being infected by COVID-19, thereby isolating more, and experiencing increased loneliness and disconnection. Meaning that even though restrictions are lifted, they are unable to participate in society on the same level as individuals without an underlying condition. This could be an explaination why we did not find the same level of relief or reduction in symptoms as in the general population in response to the softening of restrictions. If our study period lasted until the total discontinuation of restrictions, or after the vulnerable part of the population was vaccinated we might have seen different results. This is however outside the scope of our current study.

The SCL-5 and CAS measures have not specifically been tested in the rare disorders population, but rely on population samples, meaning that there are no established norms for individuals with rare disorders. To our knowledge there does not exist longitudinal studies of individuals with rare disorders across time in relation to their mental health. A future direction of studies would be to further examine how individuals with rare disorders fare in terms of mental health over time—outside of a pandemic context. This would give is important information about what we could expect from different changes.

### Strengths and limitations

A strength of the study is the unique sample of individuals and the longitudinal design with 13 assessment points. The sample-size was relatively small, which is a common limitation in research on rare disorders. However, the repeated measures design resulted in over 400 observations, increasing statistical power, and allowing us to examine longitudinal trajectories. People with rare disorders are an under-investigated group. Thus, this study provides important knowledge about a group at increased risk but that often receive limited attention. A limitation of the study is the lack of a direct comparison to the general population. Currently there are no longitudinal studies of this population outside of the COVID-19 pandemic that we know of. Furthermore, the brief measures of mental health symptoms and coronavirus anxiety can be seen as a limitation in comparison with more comprehensive clinical interviews and longer scale measures. However, brief, well-established measures with good psychometric properties can also be considered a strength, as they limited the time and effort required for participation. A coding error only made four of the five items of the *Coronavirus Anxiety scale* included in the study, limiting its reliabillity.

### Implications and conclusion

We found no systematic improvement in the levels of mental health symptoms in people with rare disorders during the COVID-19 pandemic, highlighting the importance of further investigation of the long term effects of the pandemic.

## Data Availability

The data that support the findings of this study are not openly available due to reasons of sensitivity. The data are available from the corresponding author, if you have the approval from The Regional Committee for Medical and Health Research Ethics. Data are located in a secure access data storage at The University of Oslo.
